# Bioinformatics recipes: creating, executing and distributing reproducible data analysis workflows

**DOI:** 10.1186/s12859-020-03602-6

**Published:** 2020-07-08

**Authors:** Natay Aberra, Aswathy Sebastian, Aaron P. Maloy, Christopher B. Rees, Meredith L. Bartron, Istvan Albert

**Affiliations:** 1 Department of Biochemistry and Molecular Biology, Pennsylvania State University, 201 Old Main, University Park, PA 16802 USA; 2 Northeast Fisheries Center, US Fish and Wildlife Service, Lamar, PA 16848 USA

**Keywords:** Data analysis, Scientific workflows, Reproducibility

## Abstract

**Background:**

Bioinformaticians collaborating with life scientists need software that allows them to involve their collaborators in the process of data analysis.

**Results:**

We have developed a web application that allows researchers to publish and execute data analysis scripts. Within the platform bioinformaticians are able to deploy data analysis workflows (recipes) that their collaborators can execute via point and click interfaces. The results generated by the recipes are viewable via the web interface and consist of a snapshot of all the commands, printed messages and files that have been generated during the recipe run.

A demonstration version of our software is available at https://www.bioinformatics.recipes/.

Detailed documentation for the software is available at: https://bioinformatics-recipes.readthedocs.io.

The source code for the software is distributed through GitHub at https://github.com/ialbert/biostar-central.

**Conclusions:**

Our software platform supports collaborative interactions between bioinformaticians and life scientists. The software is presented via a web application that provides a high utility and user-friendly approach for conducting reproducible research. The recipes developed and shared through the web application are generic, with broad applicability and may be downloaded and executed on other computing platforms.

## Background

The majority of bioinformatics analyses consist of several customizable computational tasks chained together to form a so-called pipeline or workflow. Publishing, documenting, and sharing these computational analyses are the cornerstones of reproducible research [1–4].

In this paper, we present a web application that allows bioinformaticians to publish and execute data analysis workflows. We call these workflows “bioinformatics recipes.” A “recipe” can be thought of as a standalone data analysis script that runs in a computing environment. A recipe may be a collection of several command-line tools, it may be a Makefile, a SnakeMake [3] file, a Nextflow [4] pipeline, an R script or any command line oriented computer program.

We designed our framework such that any series of commands may be formatted and published as a recipe. In addition, the application that we have developed can generate a graphical user interface to each recipe, thus facilitates user interaction and parameter selection at runtime.

## Implementation

Our software is a Python and Django based application that can be installed and run with minimal system administration knowledge and is aimed to be deployed to serve individual research groups.

Our software also offers project-based laboratory data management. Within the management interface, all content is grouped into projects that may have public or private visibility. Content stored in public projects is readable without restrictions. Private projects will restrict access to members only. Within each project content is divided into three main categories:

1. Data (the input files)
2. Recipes (the code that processes the data)
3. Results (the directory that contains the resulting files of applying the recipe to data)

**Fig. 1:**
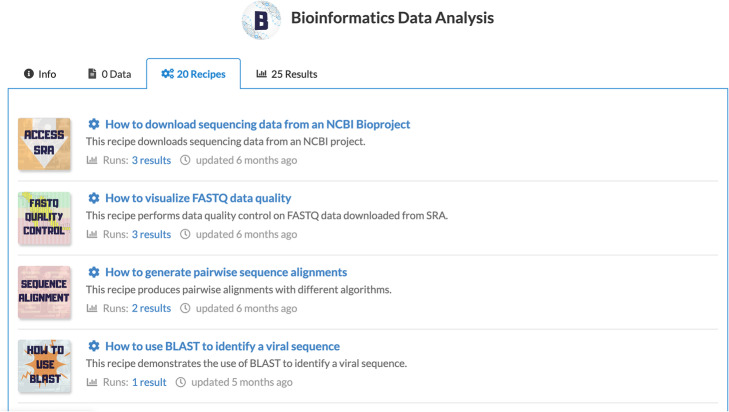
The recipe listing within a project. Each recipe has a short description as well as link to results produced by the recipe

Figure 1 shows a project view with Data, Recipes and Results displayed in separate tabs of the project. A typical workflow requires that one or more Data are combined with a Recipe to produce a Result: Data + Recipe - > Results.

First the data section must be populated. Data may be uploaded or may be linked directly from a hard drive or from a mounted filesystem, thus avoiding copying and transferring large datasets over the web. For recipes that connect to the internet to download data, for example when downloading from the Short Read Archive the data does not need to be already present in the local server.

Notably the concept of “data” in our system is broader and more generic than that for a typical file system. In our software “data” may be a single file, it may be a compressed archive containing several files or it may be a path to a directory that contains any number of files as well as other subdirectories. The programming interfaces for recipes can handle directories transparently and make it possible to run the same recipes that one would use for a single file on all files of an entire directory.

**Fig. 2:**
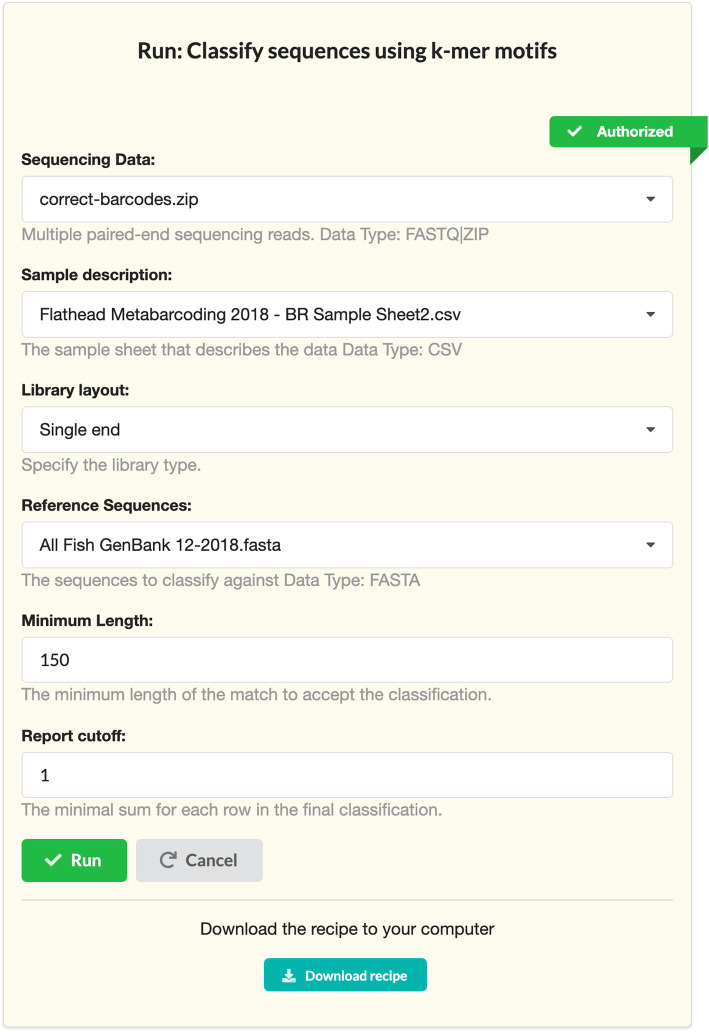
The graphical user interface for the recipe generated from the TOML specification snippet. This interface allows different parameters to be set and passed into the code of the recipe

Each recipe may be assigned a graphical user interface specification code in TOML format. From the TOML code the recipe website will generate a user interface, connected to the underlying data analysis script. For example, the TOML code (partially shown) below:

would generate the interface shown in Fig. 2. When a recipe is executed the parameters selected on the graphical user interface will replace the corresponding parameters inside the recipe. The interface generation “specification language” provides the building blocks for creating user interfaces.

**Fig. 3:**
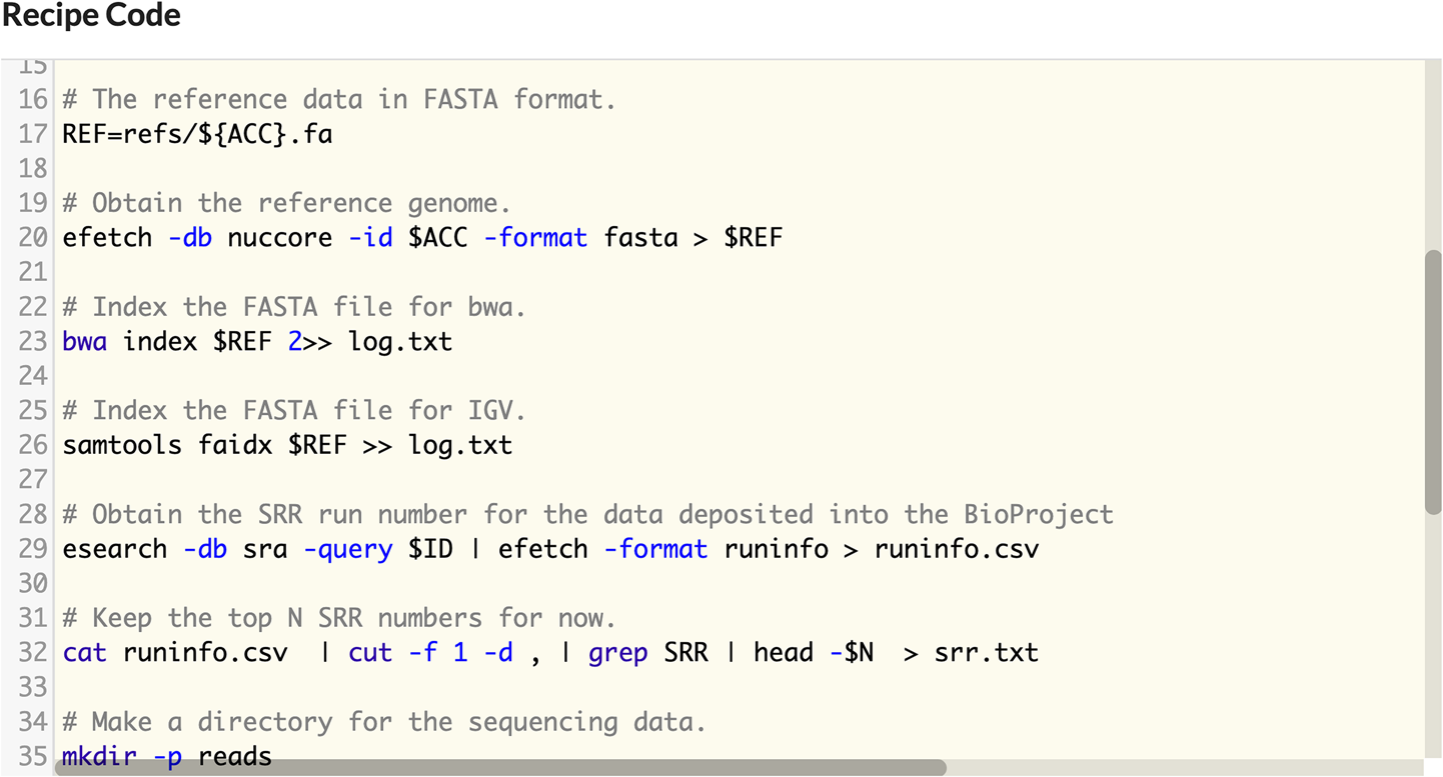
The recipe code consists of the computer code that is executed when the recipe is run. This code may be a series of shell commands, R code, Python code, or any scripting-oriented instruction set

The code for each recipe may be inspected before executing the recipe as seen in Fig. 3. Notably the recipe code consists of executable instructions that may be run on other platforms.

Running a recipe on data entry produces a “result” directory. Result directories consists of all files and all the metadata created by the recipe as it is executed on the input data. Each run of a recipe will generate a *new* result directory. Users may inspect, investigate and download any of the files generated during the recipe run. Additionally, users may copy a result file as new data input for another recipe.

**Fig. 4:**
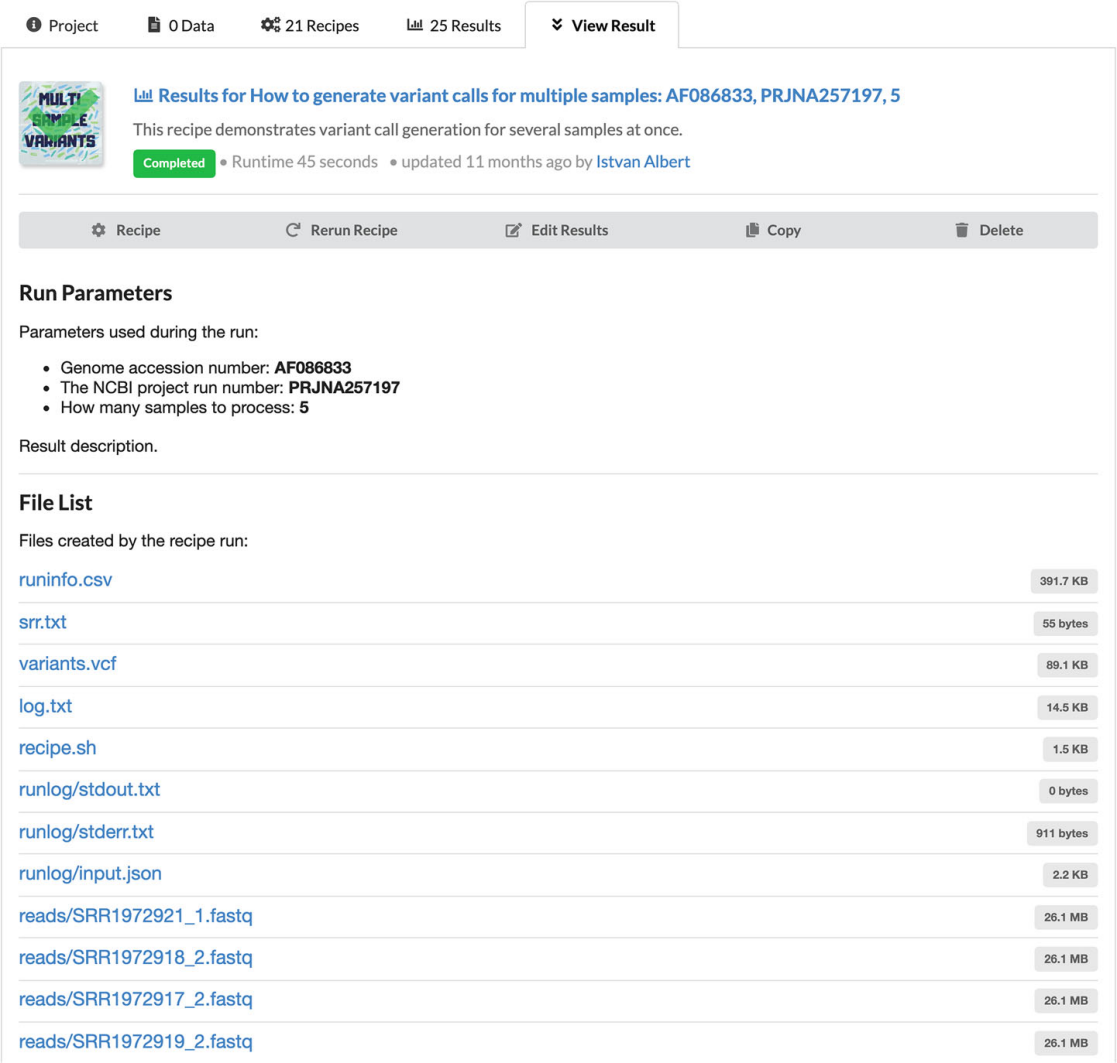
The result interface shows all the files generated by a recipe run. In addition, the directory contains all the information necessary to reproduce the analysis, the code, the metadata and a log for the standard input and output generated during the execution of the recipe

Upon executing a recipe on a dataset, a result directory is generated that lists all files created during the recipe run. See Fig. 4. In addition, all messages printed on the standard output or standard error streams are captured as files and may be inspected later.

The web application that we have developed also provides laboratory data management services. Recipes, data and results can be copied across projects, users may create new projects and may allow others (or the public) to access the contents of a project. As constructed, the web application provides a transparent and consistent framework to conduct analyses that can be shared among collaborators or with the public, and may be reproduced over time due the preservation of runtime-specific version of the code.

## Discussion

The need to simplify access to command line tools via graphical user interfaces has been long recognized by several research groups. To address this need various frameworks with similar goals [3–5] have been proposed, developed and deployed. For example, Shiny [6] is an R package that provides a framework for turning R code into an interactive webpage. Webemboss [7] was proposed as a web based environment from which a user can make use of EMBOSS tools in a user-friendly way. Our approach differs substantially from each of these prior works and is aimed to serve the needs of different audiences. Conceptually the most similar software is Galaxy [8], a web application that deploys command-line oriented bioinformatics software tools via a web based graphical user interface.

The recipe approach is similar to Galaxy in that it serves non-technical audiences. Additionally, just like Galaxy, recipes provide a user friendly, graphical interface to facilitate their use. The main difference relative to Galaxy is that, every recipe is downloadable and executable as a standalone program. Thus, recipes may be run on any computational platform and do not depend on the web interface. We refer readers to the detailed documentation of software available at https://bioinformatics-recipes.readthedocs.io/ where we discuss in detail the differences between our approach and that of existing software platforms.

Notably, in our recipe approach, the roles are more separated and distinct than in Galaxy. In our typical use cases, bioinformaticians develop and test the analysis code at the command line, then they turn their code into recipes and share them with all collaborators. Once shared via the website, collaborators can then select parameters and execute a recipe using data of their choice. Collaborators may inspect, copy, and modify the recipe code.

## Conclusion

Our software has been developed to provide bioinformatics support to metabarcoding analyses at the US Fish and Wildlife Northeast Fisheries Center and has been in operation for over a year. We found that the software is well suited for environments where bioinformaticians interact and collaborate with scientists from diverse backgrounds, and when consistent types of analyses need to be applied to varying datasets. In addition, we have found that the recipe approach integrates well into bioinformatics education. We have made use of the bioinformatics recipes website while delivering graduate level classes over several semesters at Penn State and found the approach to be well received by students. Using recipes allowed us to demonstrate the use of bioinformatics software in a manner that closely resembles their original command line usage. As instructors we were able to demonstrate complete workflows to students, show both the code and all the results that the code produced, while allowing students to copy, share and customize computational pipelines.

The current deployment contains a tutorial, education related materials as well as numerous recipes that demonstrate typical analytical workflows from quality control to RNA-Seq data analysis. We envision individual research groups and organizations running their private instances of our code to serve their local needs and audiences. Using this web platform to host the software allows the various bioinformatics analysis tools as well as the code used as part of the pipeline to be updated as new versions are available.

In conclusion, the we have developed a software that supports bioinformaticians assisting and collaborating with life scientists. The software is presented via a web application that provides a high utility and user-friendly tool for conducting reproducible research.

## Data Availability

A public deployment of the Bioinformatics Recipes software can be accessed at https://www.bioinformatics.recipes/. The website contains recipes developed for US Fish and Wildlife as well as recipes used in online courses teaching bioinformatics. The code is released with an open source license and may be accessed at: https://github.com/ialbert/biostar-central.
